# Supplementary material for: Seminoma and Embryonal Carcinoma Footprints Identified by Analysis of Integrated Genome-Wide Epigenetic and Expression Profiles of Germ Cell Cancer Cell Lines
Source: PLoS One. 2014 Jun 2;9(6):e98330. doi: 10.1371/journal.pone.0098330 (PMC4041891; doi:10.1371/journal.pone.0098330)
Supplement: File S1 — ZIP file containing DMRforPairs output for significant regions. Please start from the html files. (ZIP) [file pone.0098330.s008.zip › figures/1692.pdf]

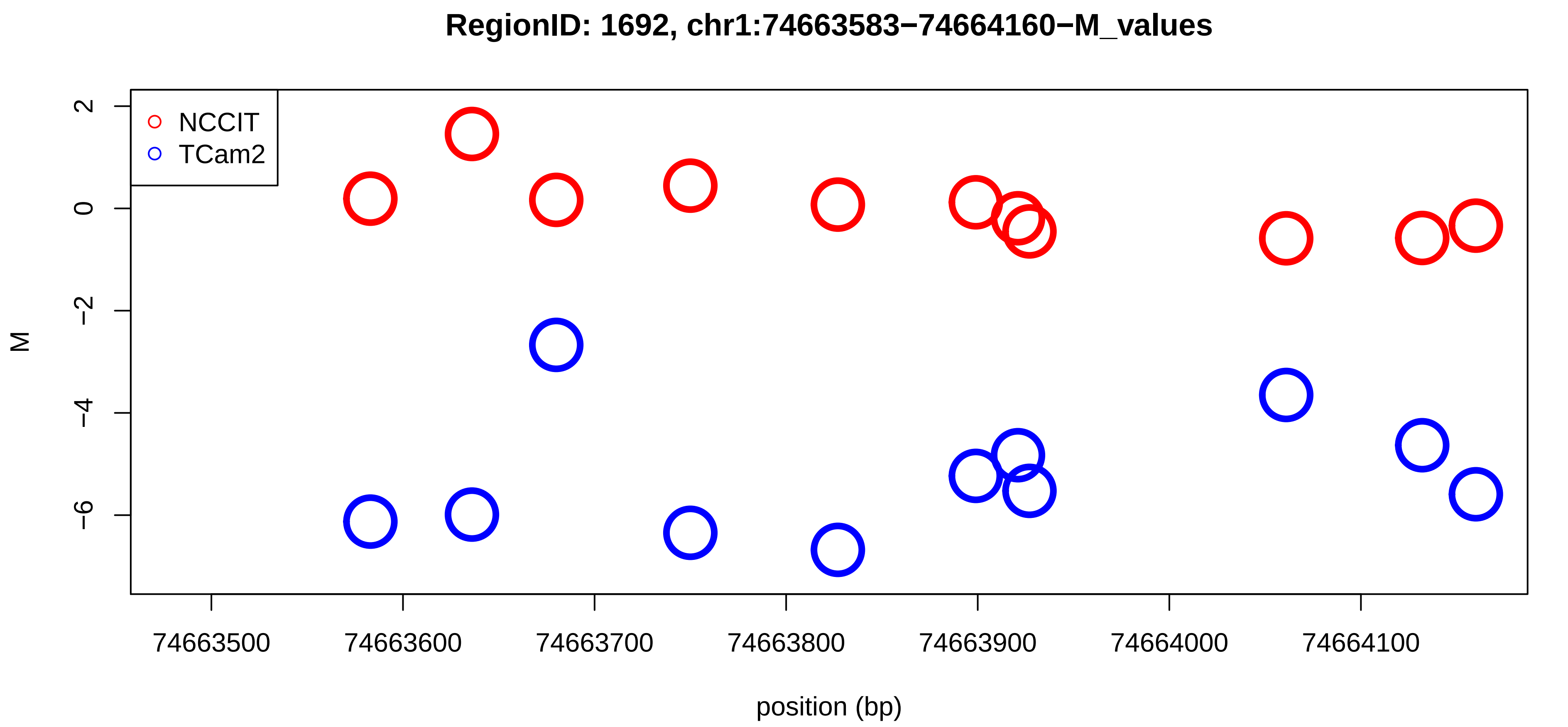

RegionID: 1692, chr1:74663583−74664160−M_values
NCCIT
TCam2
M
2
0
−2
−4
−6
74663500
74663600
74663700
74663800
74663900
74664000
74664100
position (bp)

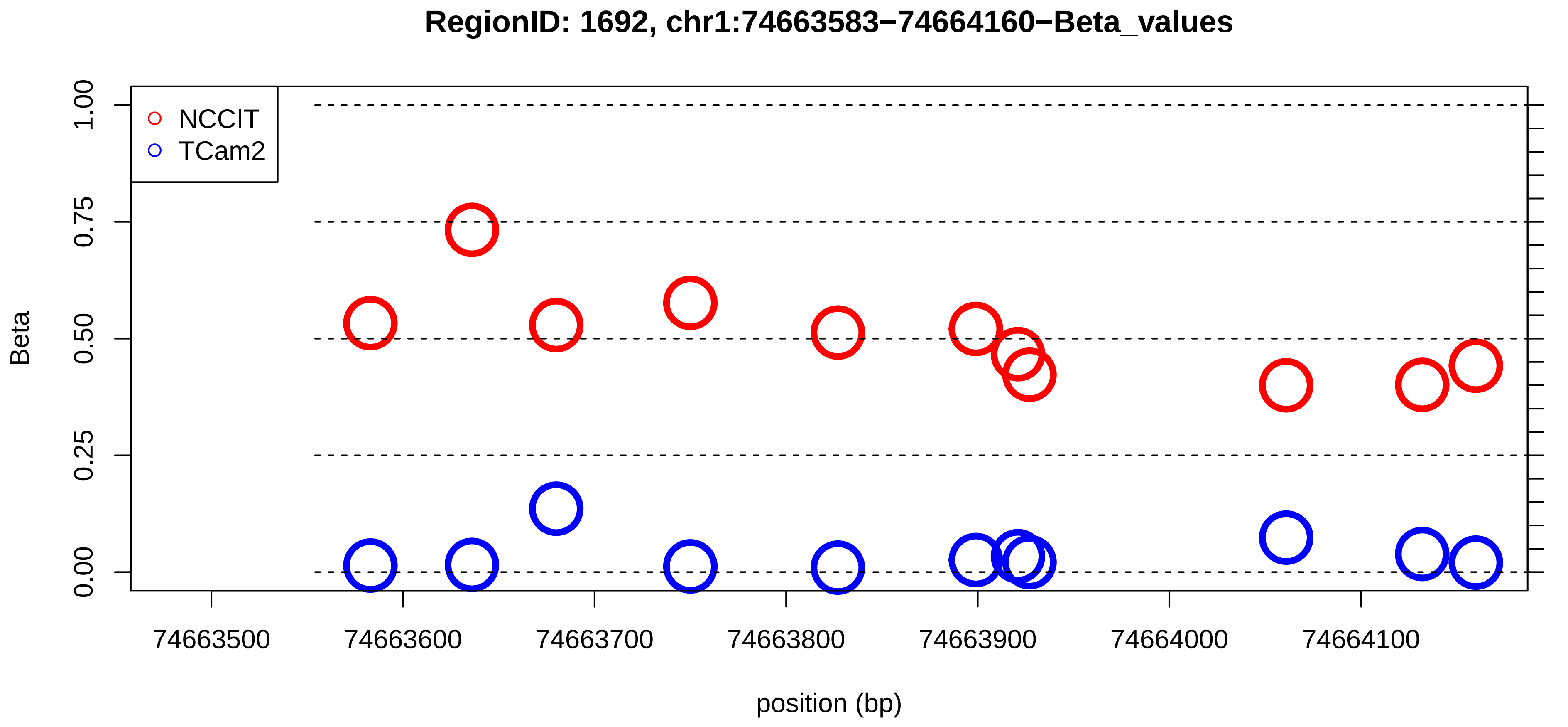

RegionID: 1692, chr1:74663583−74664160−Beta_values
NCCIT
TCam2
Beta
1.00
0.75
0.50
0.25
0.00
74663500
74663600
74663700
74663800
74663900
74664000
74664100
position (bp)

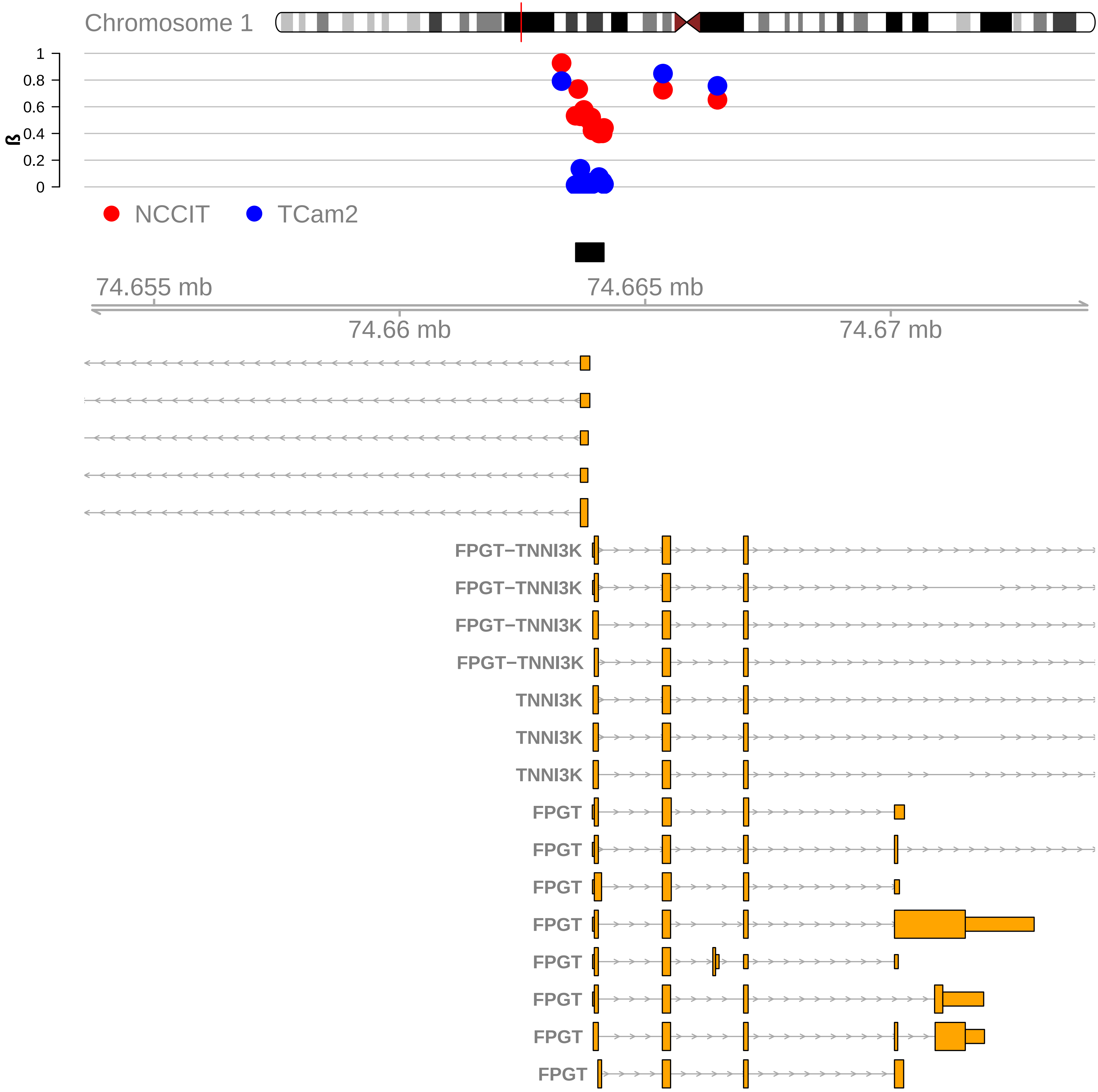

Chromosome 1
ß
1
0.8
0.6
0.4
0.2
0
NCCIT
TCam2
74.655 mb
74.665 mb
74.66 mb
74.67 mb
FPGT−TNNI3K
FPGT−TNNI3K
FPGT−TNNI3K
FPGT−TNNI3K
TNNI3K
TNNI3K
TNNI3K
FPGT
FPGT
FPGT
FPGT
FPGT
FPGT
FPGT
FPGT
